# NMR-Metabolomics Shows That BolA Is an Important Modulator of *Salmonella* Typhimurium Metabolic Processes under Virulence Conditions

**DOI:** 10.3390/metabo9110243

**Published:** 2019-10-23

**Authors:** Gil Graça-Lopes, Gonçalo Graça, Susana Barahona, Ricardo N. Moreira, Cecília M. Arraiano, Luís G. Gonçalves

**Affiliations:** 1 ITQB Nova-Instituto de Tecnologia Química e Biológica António Xavier, Universidade Nova de Lisboa, Av. da República, 2780-157 Oeiras, Portugal; gil.lopes@itqb.unl.pt (G.G.-L.); sbarahona@itqb.unl.pt (S.B.); rnmoreira@itqb.unl.pt (R.N.M.); 2 Department of Metabolism, Digestion and Reproduction, Faculty of Medicine, Imperial College London, Sir Alexander Fleming Building, London SW7 2AZ, UK; g.gomes-da-graca@imperial.ac.uk

**Keywords:** *Salmonella* Typhimurium, NMR, metabolomics, BolA, virulence

## Abstract

BolA is a ubiquitous global transcription factor. Despite its clear role in the induction of important stress-resistant physiological changes and its recent implication in the virulence of *Salmonella*, further research is required to shed light on the pathways modulated by BolA. In this study, we resorted to untargeted ^1^H-NMR metabolomics to understand the impact of BolA on the metabolic profile of *Salmonella* Typhimurium, under virulence conditions. Three strains of *S*. Typhimurium SL1344 were studied: An SL1344 strain transformed with an empty plasmid (control), a *bolA* knockout mutant (Δ*bolA*), and a strain overexpressing *bolA* (*bolA*^+^). These strains were grown in a minimal virulence-inducing medium and cells were collected at the end of the exponential and stationary phases. The extracts were analyzed by NMR, and multivariate and univariate statistical analysis were performed to identify significant alterations. Principal component analysis (PCA) and partial least squares discriminant analysis (PLS-DA) of ^1^H-NMR data allowed the discrimination between the metabolic profiles of these strains, revealing increased levels of acetate, valine, alanine, NAD^+^, succinate, coenzyme A, glutathione, and putrescine in *bolA*^+^. These results indicate that BolA regulates pathways related to stress resistance and virulence, being an important modulator of the metabolic processes needed for *S*. Typhimurium infection.

## 1. Introduction

*Salmonella enterica* is an intracellular pathogen in a myriad of animals. Among the different serotypes, *Salmonella enterica* serovar Typhimurium is one of the primary sources of gastrointestinal disease and bacteraemia in humans [1,2]. A successful infection is determined by its capacity to invade cells and replicate intracellularly, which is driven by the activity of virulence factors encoded in the *S.* Typhimurium genome [3]. These bacterial proteins are delivered to the cytosol of the host via type III secretion systems. Of particular importance are the delivery systems encoded in the *Salmonella* pathogenicity islands 1 and 2 (SPI-1 and SPI-2), which are essential for invasion and intracellular survival [4,5]. Control and coordination of all the necessary virulence elements during infection are achieved by intricate regulatory networks, of which many regulators have been described [6,7,8]. Because the virulence of *Salmonella* is, in part, determined by the ability to form biofilms and the capacity to sustain stresses, such as lack of nutrients and acidic environments, important regulators of these processes could play a significant role in the pathogenesis of *Salmonella.* One such regulator is the transcription factor BolA.

Originally found in *Escherichia coli*, BolA has been shown to be present in both prokaryotes and eukaryotes [9,10]. Initial studies of *bolA* established it as a morphogene upregulated at the onset of stationary growth phase, whose overexpression led to the acquisition of a spherical shape by cells [11,12]. It was later shown that *bolA* acted as a major stress-response gene and that its expression could be triggered during the exponential phase, if cells were exposed to carbon starvation and other stressful conditions such as oxidative, acidic, or osmotic stresses [13]. The pleiotropic effect of BolA in the cell is revealed by the important stress-resistant physiological changes that it induces. These include a decrease in the surface to volume ratio, due to changes in morphology [11], reduction of outer membrane permeability [14], and promotion of biofilm development [15,16]. In addition, the involvement of BolA in the pathogenesis of *S.* Typhimurium has been recently confirmed in a study involving the infection model *Galleria mellonella*, which showed that the production of BolA increased the virulence of this bacteria [17]. Moreover, BolA-like proteins have been widely established as key players in iron metabolism [18,19,20,21,22]. In both prokaryotic and eukaryotic organisms, BolA proteins are closely related with CGFS-type monothiol glutaredoxins (Grxs) [10,23]. Previous studies have demonstrated that BolA and monothiol Grxs form heterocomplexes involved in regulating Fe-S cluster trafficking and assembly [18,23,24,25]. *S.* Typhimurium encodes other BolA homologue, IbaG, described in *E. coli* as an acidic stress response protein [26]. Interestingly, IbaG was also shown to co-express with monothiol Grxs forming heterodimers that are involved in Fe-S assembly and trafficking, which clearly show the crucial role of BolA-like proteins in iron metabolism [27].

Despite considerable progress, further research effort is required to shed light on the pathways modulated by BolA. The use of a holistic approach, like metabolomics, could provide further insight into the matter. Metabolomics consists of the profiling and analysis of metabolites in biological samples. Its application to microorganisms can result in important knowledge, which, integrated with other “omics”, such as transcriptomics and proteomics, can provide better understanding of cell physiology [28]. Metabolomics studies of *S.* Typhimurium have had a wide range of purposes, such as studying biofilm development [29,30], discovering biomarkers for diagnostic purposes [31], or exploring the metabolic processes connected to virulence [32]. A study combining metabolomics and genome-scale modelling found an accumulation of amino acids, along with an increase in putrescine levels in *S.* Typhimurium under virulence-inducing conditions [33]. The contribution that metabolomics can provide to the understanding of pathogenesis, together with the discovery of BolA as a relevant player in the virulence of *S.* Typhimurium, led us to the objective of this work: To study the impact of BolA on the metabolic profile of *S.* Typhimurium, when grown in a virulence-inducing medium. Due to the growth-dependent regulation of *bolA*, cells were collected at the end of the exponential phase and stationary phase. ^1^H-NMR metabolomics was used to characterize the corresponding intracellular metabolomes. 

## 2. Results

### 2.1. Influence of BolA on the Growth of Salmonella Typhimurium in a Virulence-Inducing Medium

**Figure 1:**
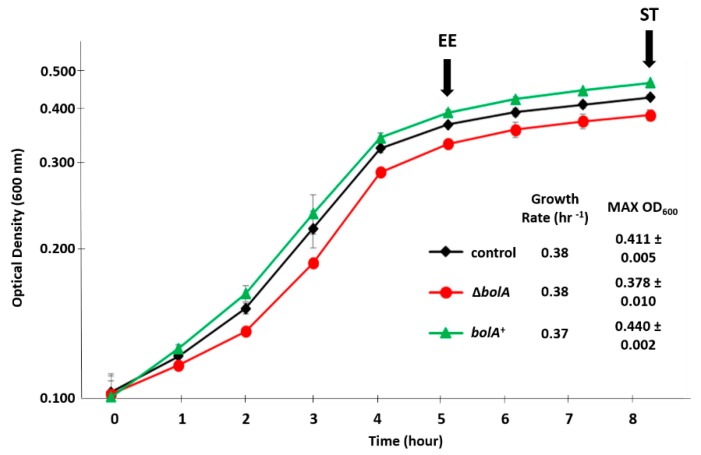
The growth curve in minimal virulence-inducing medium (LPM medium). BolA+ reaches the highest optical density, followed by the control and ΔbolA strains. The presented growth curves are the result of three independent growths. The arrows indicate the time of cells’ collection at end of exponential (EE) and stationary phase (ST).

To evaluate the effect of BolA on the growth of *Salmonella* Typhimurium, cells were grown in a minimal virulence-inducing medium (LPM medium). Initially, four strains were surveyed: A wild type strain (WT), a WT strain transformed with an empty pWSK29 plasmid (here referred as control), a *bolA* knockout mutant (Δ*bolA*), and a strain overexpressing *bolA* in pWSK29 (*bolA*^+^). The strains displayed similar growth rates (Figure 1). The WT and control strains displayed identical growth and metabolic profiles (Figure S1). Since these two strains showed the same behavior, only the control was used in the following study to guarantee that any possible effects observed on the metabolic profiles were due to the overexpression of *bolA* and not because of the pWSK29 plasmid presence.

Considering that *bolA* expression is upregulated in the late stages of growth [12], cells were collected at the end of the exponential phase (EE) and stationary phase (ST), 5 and 8 hours into growth, respectively. At these time points, cell morphology was assessed, revealing that *bolA*^+^ had acquired a spherical shape and thus confirming that this strain was overexpressing the morphogene *bolA* (Figure S2).

### 2.2. ^1^H-NMR Metabolic Profiles of Salmonella Strains

**Figure 2:**
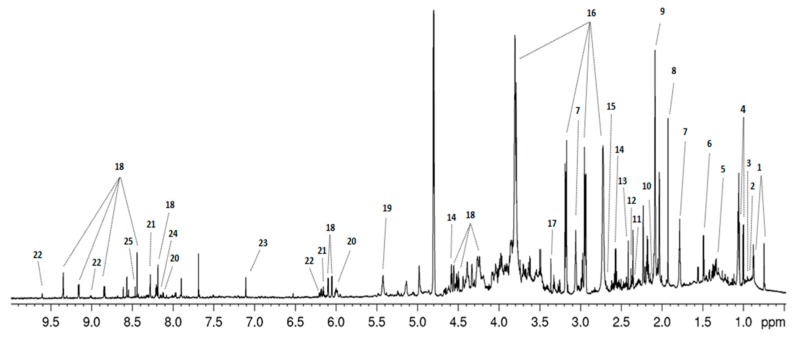
Typical ^1^H-NMR acquired with an 800 MHz spectrometer. Spectrum representative of a sample of *Salmonella* Typhimurium grown in LPM medium. Legend: 1, coenzyme A; 2, isoleucine; 3, leucine; 4, valine; 5, lactate; 6, alanine; 7, putrescine; 8, acetate; 9, glycoprotein N-acetyl groups; 10, glutamine; 11, glutamic acid; 12, pyruvate; 13, succinate; 14, glutathione; 15, methionine; 16, MES buffer (exogenous); 17, methanol (exogenous); 18, NAD^+^; 19, glycogen; 20, UMP; 21, AMP; 22, nicotinamide ribotide; 23, histidine; 24, NADP^+^; 25, formate.

In the ^1^H-NMR spectra of the intracellular extracts of *S.* Typhimurium, obtained during the ST phase, it was possible to identify 22 metabolites, 2 exogenous compounds, and protein signals, which were present in all the analyzed samples (Figure 2, Table S2). ^1^H-NMR spectra of the EE phase displayed similar profiles, though the intensity of the resonances was attenuated when compared to those of the ST phase (not shown). 

### 2.3. Multivariate and Univariate Analysis of ^1^H-NMR Data

**Figure 3:**
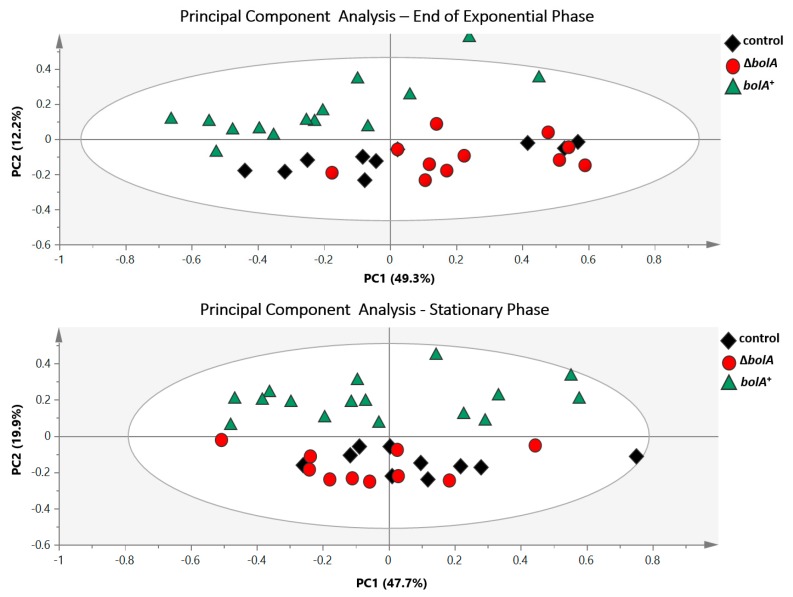
Principal component analysis (PCA) of samples representing end of exponential phase and stationary phase. The unsupervised multivariate analysis by PCA reveals a clear separation between *bolA*^+^ and the remaining strains in both phases of growth (with a R^2^X = 0.848 and R^2^X = 0.87 for the EE and ST, respectively).

**Figure 4:**
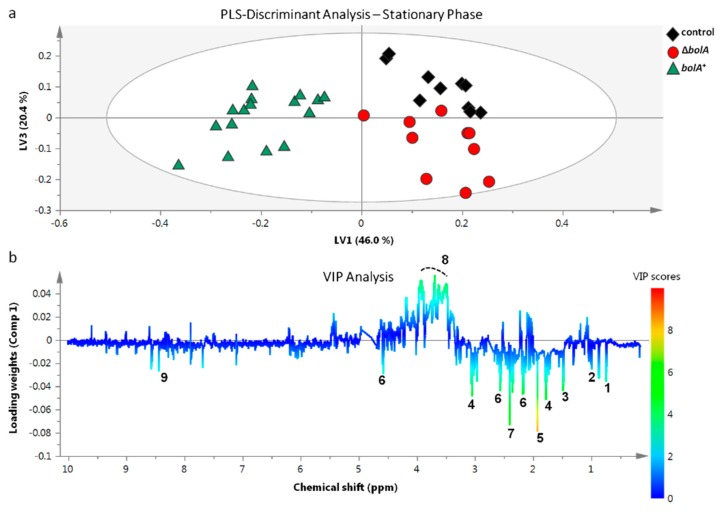
Partial least squares discriminant analysis (PLS-DA) model and variable importance in projection (VIP) analysis of the control, Δ*bolA* and *bolA*^+^ strains in the stationary phase. (**a**) Scores scatter plot of PLS-DA model of control (*n* = 10), Δ*bolA* (*n* = 10), and *bolA*^+^ (*n* = 16) samples in the stationary phase (R^2^X = 0.821, R^2^Y = 0.939, Q^2^ = 0.758). (**b**) VIP analysis of loading weights derived from component 1 of the PLS-DA model reveals the metabolites that contribute the most to the discrimination observed in the scores scatter plot. Legend: 1, coenzyme A; 2, valine; 3, alanine; 4, putrescine; 5, acetate; 6, glutathione; 7, succinate; 8, unknown compound(s); 9, NAD^+^.

To assess the existence of differences between the metabolic profiles of the control, Δ*bolA* and *bolA*^+^ strains, ^1^H-NMR data from both the EE and ST phases were analyzed resorting to principal component analysis (PCA) (Figure 3). The resulting scores plot immediately revealed a clear separation between *bolA*^+^ and the remaining strains in both phases. Therefore, the overproduction of BolA was inducing significant metabolic variation in *S.* Typhimurium. The PCA analysis also showed no separation between the control and Δ*bolA* strains. However, a supervised multivariate analysis, partial least squares discriminant analysis (PLS-DA), exposed an additional separation between the control and Δ*bolA* clusters in ST growth phase (Figure 4a). No separation between the control and Δ*bolA* groups was observed in the PLS-DA of the EE phase (Figure S3a). Permutation analysis validated the PLS-DA models of both the EE and ST growth phases, exhibiting predictive capabilities (Q^2^) of 0.599 and 0.758, respectively (Figure S4).

**Figure 5:**
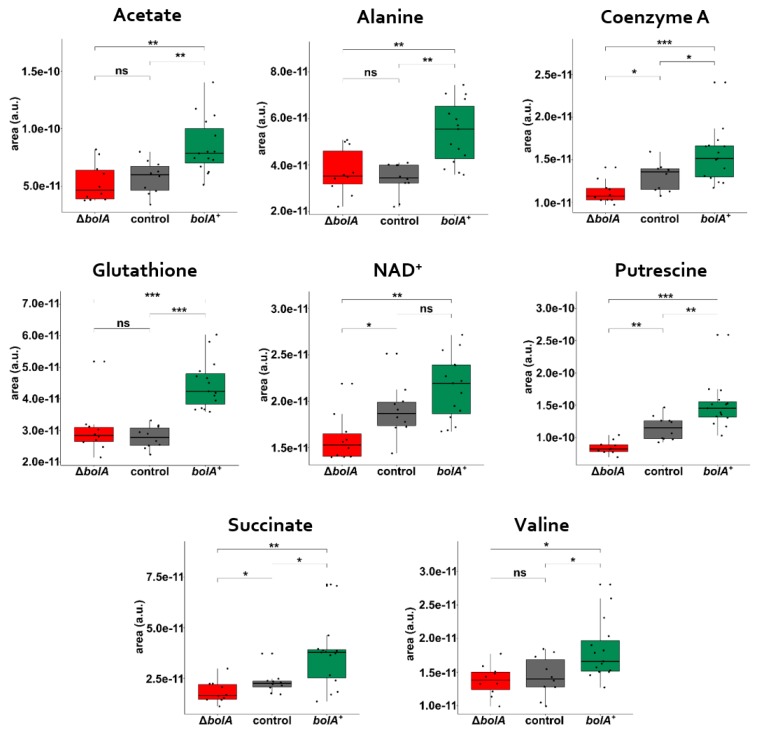
Univariate analysis of the metabolites found to be altered in the stationary (ST) growth phase. Variation of the metabolites found in the VIP analysis between the control, ΔbolA, and bolA^+^ strains. *P* > 0.05 (ns); *p* ≤ 0.05 (*); *p* ≤ 0.01 (**); *p* ≤ 0.001 (***).

The loadings resulting from the PLS-DA model, along with the implementation of the variable importance in projection (VIP), were used to estimate the significance of each metabolite in the discriminations observed in the PLS-DA scatter plots. The VIP scores of the EE growth indicated increased levels of acetate, alanine, formate, glutathione, putrescine, succinate, and valine in the *bolA*^+^ strain and increased levels of glycogen, glycoprotein N-acetyl groups, and of an unknown singlet located at 2.22 ppm in the control and Δ*bolA* groups (Figure S3b). The VIP analysis of loading weights derived from component 1 of the ST growth phase revealed that *bolA*^+^ could be separated from the remaining strains based on differences in acetate, alanine, coenzyme A, glutathione, NAD^+^, putrescine, succinate, and valine, which were increased in the *bolA*^+^ strain (Figure 4b). In addition, VIP scores derived from component 3 of the ST phase allowed discrimination between the control and Δ*bolA* (Figure S5). In both growth phases, unidentified NMR resonances at 3.4 and 4.0 ppm, had higher levels in the control and Δ*bolA* groups. These metabolic variations were further evaluated by univariate analysis, which exposed the metabolic variations observed in EE phase as nonsignificant, but validated the altered metabolites found in the ST phase. (Figure S6 and Figure 5). Acetate, alanine, coenzyme A, glutathione, NAD^+^, putrescine, succinate, and valine were found to be significantly altered in the *bolA*^+^ strain during the ST growth phase. 

## 3. Discussion

BolA is a global transcription factor capable of inducing significant physiological changes in *Escherichia coli*. Its overproduction induces round morphology, promotion of biofilm development, and a decrease in the permeability of the outer membrane [11,13,14]. Although *bolA* is a well-established stress-response gene in *E. coli*, the role of its homologue in *Salmonella* Typhimurium is only starting to be assessed. In the infection model *Galleria mellonella*, the deletion of *bolA* led to lower intracellular replication and consequent decrease in the virulence of *S.* Typhimurium [17]. To further understand how BolA might be modulating the metabolism of *S.* Typhimurium in a virulence scenario, we sought to use ^1^H-NMR metabolomics to explore the metabolic variations between different strains expressing *bolA* in a minimal virulence-inducing medium (LPM medium).

The impact of BolA on the metabolic signature of each strain was assessed by analysis of the ^1^H-NMR spectra. Independently of being the EE or the ST phase, principal component analysis (PCA) of ^1^H-NMR data disclosed identical results. The evident discrimination between *bolA*^+^ and the remaining two strains implied that BolA overproduction significantly altered the metabolic profile of *S.* Typhimurium. Together with the existence of a cluster containing the control and Δ*bolA* strains, PCAs suggest that the overexpression of *bolA* has a greater effect on cell metabolism, when compared to the deletion of this gene.

Partial least squares discriminant analysis (PLS-DA) of the EE phase showed similar results to those of the corresponding PCA, whereas the model obtained for the ST phase exposed the control and Δ*bolA* groups as having distinct metabolic profiles (Figure 4a). These results indicate that while at the onset of stationary phase the expression of *bolA* in the control strain might have minimal influence on cell metabolism, the upregulation of this morphogene as conditions become more strenuous leads to significant metabolic changes.

Loadings derived from both models were subjected to a variable importance in projection (VIP) analysis, which allied to additional validation by univariate analysis allowed the identification of the metabolites whose levels varied between strains in the EE and ST data sets. Analysis of the EE phase suggested important metabolic variations between *bolA*^+^ and the control and Δ*bolA* groups. These included altered levels of 10 metabolites and of a signal characteristic of *N*-acetyl groups of glycoproteins. However, further evaluation by univariate analysis exposed most of these metabolic variations as nonsignificant (Figure S5). A closer look into some of the metabolites involved in these nonsignificant comparisons, such as succinate, valine, or formate, show a strong tendency for variation between strains. Yet, data dispersion in the same condition, as seen by the outliers and long ranges of the plots, could be contributing to the absence of significant statistical relationship between the analyzed variables. The greater metabolic and physiological changes associated with the EE growth phase transition could be responsible for the high variation observed in samples, turning metabolomics analysis of this phase more prone to error than the more stable ST phase. Nonetheless, putrescine was clearly increased in *bolA*^+^ and significant variation was present in metabolites, such as alanine and glutathione, which are decreased in the control strain. 

Analysis of the loadings derived from the PLS-DA of the ST phase led to the identification of eight significantly altered metabolites, which shared the peculiarity of being increased in *bolA*^+^ compared to the other strains. These metabolites included cofactors (coenzyme A and NAD^+^), amino acids (alanine and valine), acetate, glutathione, putrescine, and succinate. Of these, coenzyme A, NAD^+^, putrescine, and succinate were not only significantly increased in *bolA*^+^ relatively to the remaining strains, but also increased in the control strain when compared to Δ*bolA*, suggesting a positive correlation between the expression of *bolA* and the levels of these metabolites in the cell.

The peptidoglycan layer, located in the periplasmic space of gram-negative bacteria, is responsible for maintaining cell shape, and amino acids such as D-alanine and D-glutamic acid are essential components of this polymer [34]. Peptidoglycan insertion is regulated by MreB, a eukaryotic actin homologue responsible for rod-shape maintenance. Chemical inactivation of *mreB* has been shown to induce a round morphology in *Salmonella* [35]. Interestingly, BolA overproduction induces a spherical morphology and high levels of this protein have been shown to reduce the levels of MreB in *Escherichia coli* [36]. Additionally, production and release of D-amino acids into the growth medium have been reported during the stationary phase in a variety of bacterial species. The specific D-amino acids identified in these secretions varied between species, but these molecules seemed to regulate cell wall synthesis and composition [37]. Finally, in a previous study, it was also shown the importance of BolA in the peptidoglycan biosynthesis pathways [38]. Thus, we propose that in *S.* Typhimurium the observed increase in the intracellular pools of valine and alanine could be the consequence of an intricate network involving BolA and MreB, culminating in a process of peptidoglycan remodeling during stationary phase.

The increased levels of acetate could be linked to overflow metabolism. This process occurs when acetyl-coenzyme A is directed to mixed acid fermentation, with consequent production of acetate. It has been shown that the energy necessary for the replication of *S.* Typhimurium, in some epithelial and macrophage cell lines, is generated primarily by overflow metabolism to acetate and lactate [32]. In addition, acetate has been found to promote SPI-1 expression [39]. The link between these mechanisms and virulence, together with the overexpression of *bolA*, strengthen the role of this gene as a promotor of *Salmonella* pathogenesis.

A positive correlation seems to exist between the levels of succinate, coenzyme A, and NAD^+^ and the expression of *bolA* (Figure 5). NAD^+^ is a critical oxidizing agent in key steps of both glycolysis and citric acid cycle (CAC) and increased concentration of this cofactor in the cytosol promotes glycolysis [40]. Given the pivotal role of NAD^+^, coenzyme A, and succinate in the central metabolism, increased levels of these metabolites must indicate higher glycolytic and CAC fluxes.

The increased levels of glutathione and putrescine in the *bolA*^+^ are particularly interesting, as these molecules are known to confer protection to a variety of stresses. Glutathione provides resistance to osmotic and oxidative stresses, as shown by the role of this tripeptide in the osmoadaptation of *E. coli* and the resistance to hydrogen peroxide during the stationary phase [41,42]. Another important role of glutathione is in the cell iron homeostasis and the protection of the Fe-S proteins, which interconnects with the regulatory role of BolA-like proteins in iron metabolism [25,42,43]. The positive correlation between BolA and the glutathione levels in *Salmonella* indicates that BolA have an important role in iron homeostasis in this organism that should be further studied. Putrescine was already reported in a metabolomics study as being accumulated in virulence-inducing conditions [33]. Putrescine is a polyamine whose synthesis can occur either by conversion of agmatine or conversion of L-ornithine. Polyamines have been reported to decrease the permeability of the outer membrane in *E. coli* by binding to the OmpC and OmpF porins [44]. It has also been previously reported that the overexpression of *bolA* leads to a permeability decrease of the outer membrane by upregulating the production of OmpC, a less permeable porin compared to OmpF [14]. Thus, the mechanisms by which polyamines and BolA decrease OM permeability are distinct. Nonetheless, as a transcription factor with pleiotropic effects in the cell, BolA could participate in the regulation of two different mechanisms with the same goal. Polyamines have also been shown to be essential in the case of nitrosative stress. Inhibition of growth has been documented in mutants of *S.* Typhimurium for polyamine biosynthesis, upon addition of reactive nitrogen species (RNS) to the growth medium. Genetic complementation of the *speB* gene, needed for putrescine biosynthesis, restored normal growth [45]. RNS, such as nitric oxide, have antimicrobial roles and are employed by infected macrophages as a weapon against intracellular pathogens [46]. Higher levels of putrescine, brought about by the overproduction of BolA, could thus contribute to the resistance to RNS in the context of infection. Of additional importance is the implication of polyamines as essential for the virulence of *S.* Typhimurium. Both the capacity to invade epithelial cells and intracellular replication were reported to significantly decrease in mutants of *S.* Typhimurium for polyamine biosynthesis. Further experiments revealed decreased levels of expression of SPI-1 and SPI-2 genes, necessary for the processes of invasion and intracellular survival. Moreover, invasion was slightly enhanced and replication significantly improved by complementation with the *speB* gene [47]. Interestingly, a later study revealed that spermidine, a polyamine that derives from putrescine, plays an important role in intracellular replication of *S.* Typhimurium [45]. BolA has been recently discovered to contribute to the intracellular survival of *S.* Typhimurium during infection, thereby promoting virulence [17]. An association between BolA and polyamine biosynthesis may thus exist. A plausible hypothesis is the regulation of *speB* or of a gene further upstream in the biosynthetic pathway of putrescine.

## 4. Materials and Methods

### 4.1. Bacterial Strains and Plasmids

All strains and plasmids used in this work are indicated in Table S1. All *Salmonella* strains are isogenic of *S.* Typhimurium strain SL1344 [48]. The *bolA* knockout mutant strain (Δ*bolA*) was described in a previous study [17]. To obtain the strain overexpressing *bolA* (*bolA*^+^), the pRMA04 plasmid was used to transform electrocompetent SL1344 cells. The control strain was obtained by transforming SL1344 electrocompetent cells with the backbone plasmid pWSK29.

### 4.2. Growth Conditions

For metabolomics experiments, cells were grown overnight in lysogeny broth (LB) medium, consisting of 1% sodium chloride, 1% tryptone, and 0.5% yeast extract, at 37 °C and 220 rpm. Afterwards, cells were transferred to a minimal and virulence-inducing, low phosphate, low magnesium medium (LPM), consisting of 0.5 µM ammonium ferric citrate, 8 µM MgCl_2,_ 7.5 mM (NH_4_)_2_ SO_4_, 0.5 mM K_2_SO_4_, 5 mM KCl, 0.4% glucose (w/v), 0.00001% thiamine (w/v), 337 µM H_3_PO_4_, and 80 mM 2-(N-morpholino) ethanesulfonic acid (MES) at pH 5.8 [33]. Since *Salmonella* Typhimurium SL1344 is auxotroph for histidine, LPM medium was supplemented with 257.8 µM of histidine. Antibiotics were used with the following concentrations: 90µg/mL streptomycin, 25µg/mL chloramphenicol, and 100 µg/mL ampicillin or its analogue carbenicillin. 

### 4.3. Sample Preparation for Metabolomics Analysis (NMR)

**Figure 6:**
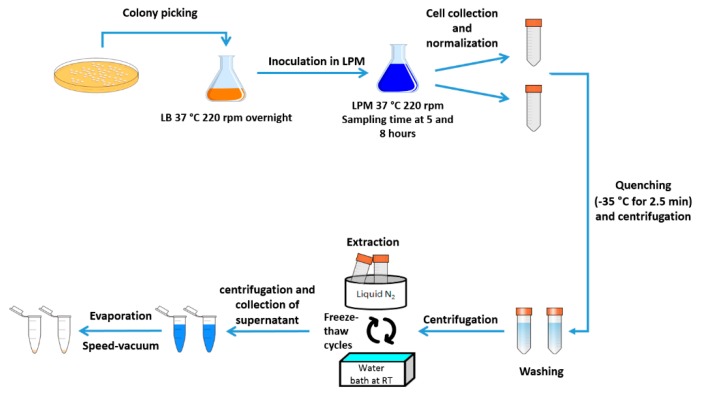
Scheme of the protocol for metabolite extraction of *Salmonella* Typhimurium. Cells were inoculated in lysogeny broth (LB) medium and incubated overnight. Following the inoculation in LPM medium, cells were harvested at specific time points, quenched at −35 °C and centrifuged. Cell pellets were washed and resuspended in an extraction solvent consisting of methanol/chloroform/water (3:1:1 v/v). After three freeze-thaw cycles, the extracted samples were centrifuged, their supernatants collected and evaporated on a speed-vacuum concentrator.

Single colonies of each strain (WT, ∆*bolA, bolA*^+^, and WT-pWSK29) were taken from an agar plate, transferred to 20 mL of LB, and incubated overnight at 37 °C, 220 rpm. The overnight cultures were centrifuged at 4000 *g* for 10 min at 4 °C. The supernatants were discarded; cell pellets were washed in 15 mL of LPM (4 °C) and centrifuged at 4000 *g* for 10 min at 4 °C [33]. The supernatants were discarded and cell pellets were resuspended, for a second time, in 15 mL of LPM (4 °C). The optical densities at 600 nm (OD_600_) were measured and used to inoculate 350 mL of LPM medium at an initial OD_600_ of 0.1. Each 350 mL culture was split into three volumes of 100 mL (two volumes for metabolite extraction at two distinct points of growth; one volume to measure OD_600_ along growth) and incubated at 37 °C, 220 rpm. Sample collection occurred at two points in time: 5 hours (end of exponential phase) and 8 hours (stationary phase) after inoculation (0 hours). For each time point, 2 × 45 mL of culture, corresponding to the strain with the lowest measured OD_600_ (Δ*bolA*), were collected. The volumes collected for the remaining strains were normalized according to the OD_600_ of Δ*bolA*. Once cells were harvested, these were immediately transferred to a bath at −35 °C (JULABO FP50-HE) for 2.5 min. The suspensions were centrifuged at 3600 *g* for 8 min at 4 °C and the supernatants were discarded. Cell pellets were washed in 40 mL of 1 × phosphate buffer (4 °C, pH 5.8) and centrifuged at 3600 *g* for 8 min at 4 °C. After supernatants were discarded, cell pellets were suspended in 900 µL of an extraction solvent consisting of methanol/chloroform/water (3:1:1, −80 °C) (EMSURE® Merck) and submitted to three freeze-thaw cycles using liquid nitrogen [49]. The extracted samples were centrifuged at 12,900 *g* for 10 min at 4 °C. The supernatants were collected and the extraction was performed a second time. The resulting samples (1.8 mL) were concentrated using a speed-vacuum concentrator (Savant^TM^). A schematic representation of the metabolite extraction protocol can be seen in Figure 6. Dried samples were dissolved in 572 µL of D_2_O and 116.5 µL of potassium phosphate buffer 0.35 M at pH 7.0 in D_2_O with 2 mM NaN_3_. Then, 11.5 µL of 2.95 mM in D_2_O were added, adding up to a total of 700 µL. Subsequently, 600 µL of each sample were transferred to a 5 mm NMR tube.

### 4.4. NMR Acquisition 

NMR spectra were acquired on a Bruker Avance II+ 800 MHz spectrometer equipped with a 5 mm TXI-Z H/C/N/-D probe. All 1D ^1^H were acquired at 298.15 K and using a *noesygppr1d* pulse program, in which water presaturation occurred during mixing time and relaxation delay. Acquisition parameters are the following: 256 scans, relaxation delay of 4 s, mixing time of 10 ms, spectral width of 16,025.641 Hz. Size of free induction decay (FID) was 128k points. Processing of spectra was performed with Bruker TopSpin 3.2. All FID were multiplied by an exponential function, followed by Fourier Transformation. Spectra were manually phased and baseline corrected. Chemical shifts were adjusted according to the chemical shift of trimethylsilylpropanoic acid (TSP) at 0.00 ppm. For the purpose of spectral assignment, 2D NMR spectra were acquired for some samples: ^1^H-^1^H TOCSY (*dipsi2esgpph* pulse sequence, 512 points in F1 and 2048 points in F2; 128 scans; relaxation delay of 1.5 s; mixing time of 60 ms; sweep width of 8012.12 Hz for both dimensions); ^1^H-^13^C HSQC (*hsqcetgpsisp2* pulse sequence, 512 points in F1 and 2048 points in F2; 128 scans; relaxation delay of 1.5 s; sweep width of 33,207.441 Hz in F1 and 12820.513 Hz in F2) and ^1^H *J*-resolved (*jresgpprqf* pulse sequence, 100 points in F1 and 8192 points in F2; 16 scans; relaxation delay of 2 s; sweep width of 78.113 Hz in F1 and 133,68.984 in F2).

### 4.5. H NMR Data Analysis

All processed 1D ^1^H NMR spectra were grouped and transformed into a matrix (each column was a spectrum, each row was one of the 128k points that makes up the FID). The spectra were aligned and centered according to the chemical shift of TSP at 0.00 ppm. The region of water (4.70–4.95 ppm) was removed, as well as peaks resulting from exogenous compounds: MES (2.670–2.770 ppm; 2.910–2.955 ppm; 3.152–3.195 ppm; 3.740–3.82 ppm) and methanol (3.34–3.38 ppm). After trimming the interfering peaks, only the region between 0.15–10.00 ppm was used. A binning of 8 points was conducted, for the purposes of data reduction, and spectra were normalized by total spectral intensity. All the aforementioned steps were completed using R software environment for statistical computing (version 3.3.2). Principal component analysis (PCA) and partial least squares discriminant analysis (PLS-DA) were performed using SIMCA 13.0.3. The 7-fold cross-validation method was used to evaluate the goodness of prediction (Q^2^) value of the resulting models, with a permutation analysis to further confirm the validity of the PLS-DA model. Briefly, the spectral matrix was divided into 7 blocks. In every cross-validation round, one block is left out (validation set) and a model is generated with the remaining spectra (training spectra). The cross-validation parameters were obtained by predicting the class of the samples in the validation set. The process was then repeated 7 times to derive the final cross-validation parameters of the model. To understand which metabolites were contributing the most to the separation observed in the PLS-DA, a variable importance in projection (VIP) analysis was conducted using the loadings derived from the PLS-DA. The metabolites responsible for the discrimination between groups of samples were also analyzed by univariate analysis. Briefly, the areas of the peaks corresponding to these metabolites were integrated and normalized by the total intensity of the corresponding spectrum. Pairwise comparisons were conducted using the Wilcoxon rank-sum test to look for significant differences (p < 0.05) between the groups of samples. Correction for multiple testing was performed using the Benjamini-Hochberg procedure. 

### 4.6. Microscopy Analysis

For microscopy analysis, the strains WT-pWSK29, Δ*bolA*, and *bolA*^+^ were grown, independently, in 50 mL of LPM medium. Cells were collected at 4 different time points: 1, 3, 5, and 8 hours. Once collected, cells were spun at 3600 *g* for 8 min and suspended in 100 µL of fresh LPM medium. Cells were observed with a phase-contrast microscope (Leica DM6000B) with a 100x oil objective.

## 5. Conclusions

Our data strengthens the role of *bolA* as an important stress-response gene and provides further evidences of BolA role as an enhancer of virulence in *Salmonella* Typhimurium. The overexpression of the *bolA* gene, under virulence-inducing conditions, led to increased pools of eight metabolites: Acetate, valine, alanine, NAD^+^, succinate, coenzyme A, glutathione, and putrescine, all of which are involved in pathways related to stress resistance and virulence. Further studies are warranted to shed more light into the metabolic pathways affected by BolA overexpression in *S.* Typhimurium in relation to virulence. However, our data support BolA as an important player of the metabolic remodeling in the virulence process of *S.* Typhimurium.

## Data Availability

The metabolomics and metadata reported in this paper are available via http://www.itqb.unl.pt/~lgafeira/Salmonella_data/.

## Supplementary Materials

The following are available online at https://www.mdpi.com/2218-1989/9/11/243/s1, Table S1: Strains used in the study, Table S2: Metabolites identified in the study, Figure S1: Comparison between the WT strain and the control strain, Figure S2: Microscopic pictures of the different strains, Figure S3: PLS-DA model at end of the exponential phase, Figure S4: Validation of the PLS-DA models, Figure S5: Metabolites variation at end of the exponential phase.
