# Supplementary material for: NMR-Metabolomics Shows That BolA Is an Important Modulator of Salmonella Typhimurium Metabolic Processes under Virulence Conditions
Source: Metabolites. 2019 Oct 23;9(11):243. doi: 10.3390/metabo9110243 (PMC6918366; doi:10.3390/metabo9110243)
Supplement: Supplementary file 1 [file metabolites-09-00243-s001.pdf]

Electronic Supplementary Material

NMR-Metabolomics shows that BolA is an important modulator of S. Typhimurium metabolic processes under virulence conditions.

Gil Graça-Lopes, Gonçalo Graça, Susana Barahona, Ricardo N. Moreira, Cecília M. Arraiano*, Luís G. Gonçalves*

ITQB NOVA, Instituto de Tecnologia Química e Biológica António Xavier, Universidade Nova de Lisboa, Portugal

Corresponding authors:
Luís G. Gonçalves
ITQB NOVA, Universidade Nova de Lisboa
Av da República-EAN
2780-157 Oeiras
Portugal
Email:lgafeira@itqb.unl.pt; Tel:+351214469561

Cecília M. Arraiano
ITQB NOVA, Universidade Nova de Lisboa
Av da República-EAN
2780-157 Oeiras
Portugal
Email:cecilia@itqb.unl.pt; Tel:+351214469547

Table S1
Description of the strains and plasmids used in this work.

Strain	Name used in the paper	Genotype	Source or reference
SL1344	WT	*Str^r hisG rpsL xyl*	(Hoiseth and Stocker 1981)
CMA820	Δ*bolA*	SL1344 Δ*bolA*::Cat^r	(Mil-Homens et al. 2018).
CMA822	*bolA*^+	SL1344 carrying pRMA04 plasmid	This study
CMA823	control	SL1344 carrying pWSK29	This study

Plasmids	Description	Origin/marker
pWSK29	Low copy plasmid	pSC101/Amp^r
pRMA04	pWSK29 encoding *bolA*	pWSK29/Amp^r

Table S2

List of metabolites identified by 1H-NMR in the intracellular environment of *Salmonella* Typhimurium grown in a virulence-inducing medium. ChEBI ID code and chemical shifts provided. s – singlet; d – doublet; dd – doublet of doublets; t – triplet; m – multiplet; bs – broad signal.
The NMR spectra used in this work is available at:
http://www2.itqb.unl.pt/~lgafeira/external/Salmonella_data/

Number	Metabolite	ChEBI ID	Assignments ppm (group, multiplicity)	MSI Level
1	coenzyme A	15346	0.73 (CH_3, s), 0.85 (CH_3, s), 2.46 (CH_2, t), 6.16 (CH, d), 8.25 (CH, s), 8.54 (CH, s)	2
2	L-isoleucine	17191	0.93 (CH_3, t), 1.00 (CH_3, d), 1.25 (CH_2, m), 1.97 (CH, m)	2
3	L-leucine	15603	0.95 (CH_3, t), 1.70 (CH, m), 1.7 (CH_2, m)	2
4	L-valine	16414	0.98 (CH_3, d), 1.03 (CH_3, d), 2.26 (CH, m)	2
5	lactate	24996	1.31 (CH_3, d), 4.10 (CH, q)	2
6	L-alanine	16977	1.47 (CH_3, d), 3.77 (CH, dd)	2
7	putrescine	17148	1.76 (CH_2, m), 3.04 (CH_2, m)	2
8	acetate	30089	1.92 (CH_3, s)	2
10	L-glutamine	18050	2.13 (CH_2, m), 2.44 (CH_2, m), 3.76 (CH, m)	2
11	L-glutamic acid	16015	2.12 ($CH_{2,}$ m), 2.34 ($CH_{2,}$ m)	2
12	pyruvate	15361	2.36 (CH_3, s)	2
13	succinate	26806	2.39 (CH_2, s)	2
14	glutathione	16856	2.15 (CH_2, m), 2.55 (CH_2, m), 2.96 (CH_2, dd)	2
15	L-methionine	16643	2.13 (CH_3, s), 2.12 (CH_2, m), 2.63 (CH_2, t)	2
16	MES buffer	39005	2.69 (CH_2, s), 2.92 (CH_2, m), 3.16 (CH_2, m), 3.77 (CH_2, m)	2
17	methanol	17790	3.34 (CH_3, s)	2
18	NAD^+	15846	4.42 (CH, dd), 4.54 (CH, m), 6.03 (CH, d), 6.08 (CH, d), 8.19 (CH, m), 8.42 (CH, s), 8.82 (CH, d), 9.14 (CH, d), 9.33 (CH, s)	2
19	glycogen	28027	3.65 (CH, m), 5.41 (CH, s)	2
20	uridine 5'-monophosphate	16695	4.41 (CH, t), 5.97 (CH, d), 5.99 (CH, d), 8.10 (CH, d)	2
21	adenosine 5’-monophosphate	16027	4.50 (CH, dd), 6.13 (CH, d), 8.25 (CH, s), 8.59 (CH, s)	2
22	Nicotinamide ribotide	16171	4.61 (CH, m), 4.65 (CH, t), 6.19 (CH, d), 8.31 (CH, dd), 8.99 (CH, d), 9.33 (CH; d), 9.58 (CH, s)	2
23	L-histidine	15971	3.14 (CH_2, dd), 3.23 (CH_2, dd), 7.09 (CH, d), 7.88 (CH, d)	2
24	$NADP^+$	18009	6.09 (CH, d), 8.14 (CH, s), 8.19 (CH, dd), 8.41 (CH, s), 8.83 (CH, d), 9.10 (CH, d), 9.29 (CH, s)	2
25	formate	15740	8.44 (CH, s)	2
	**Lipid and protein signals**		**Assignments: ppm (group, multiplicity)**	
9	glycoprotein-related		2.06 (CH_3 N-acetyl groups, bs)	3

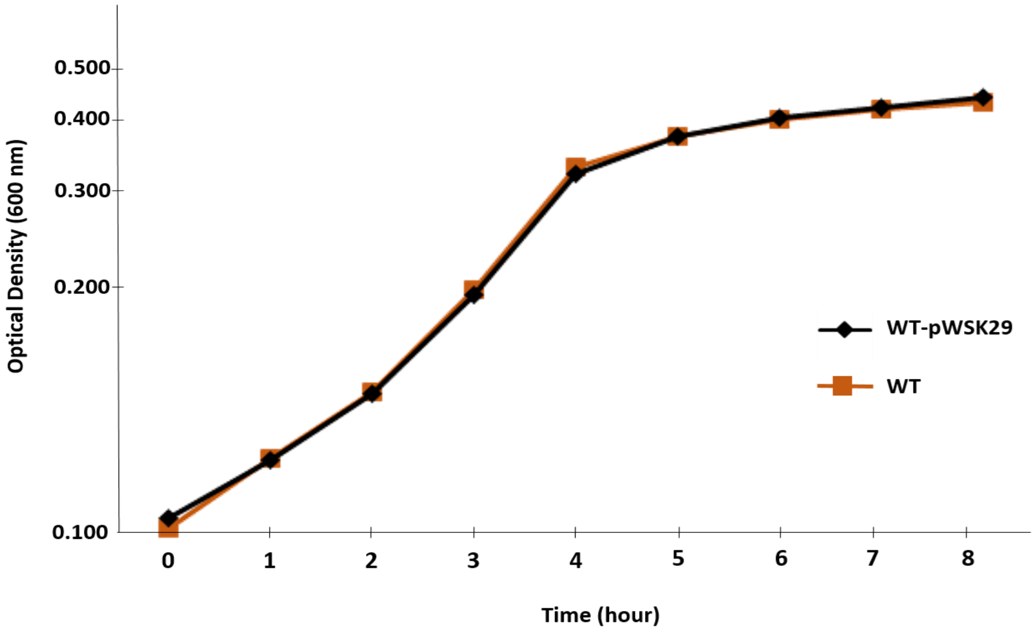

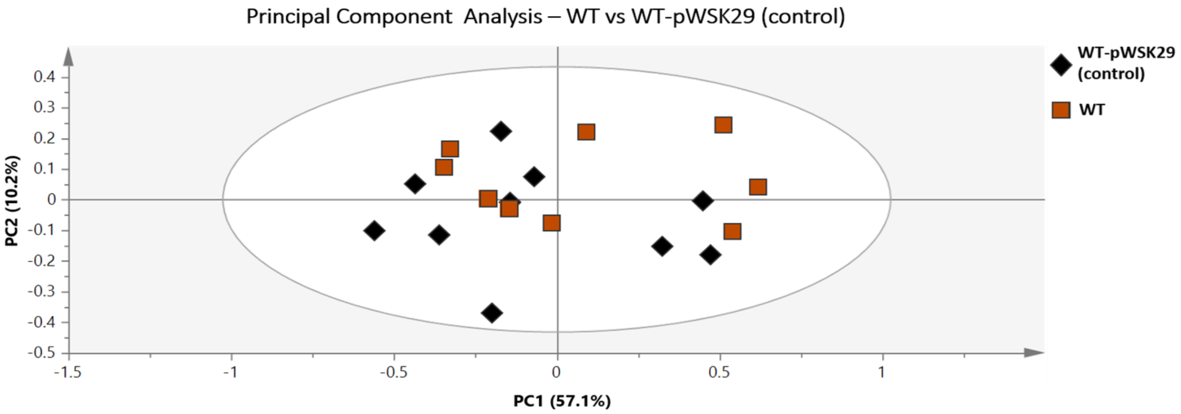

Fig. S1

Growth curve and PCA analysis of the strains WT and WT-pWSK29. **a)** WT and WT-pWSK29 exhibit similar growth profiles. **b)** The unsupervised multivariate analysis failed to reveal a shift in the normal metabolism of SL1344, upon addition of the pWSK29 plasmid, allowing no discrimination between the metabolic profiles of the two strains.

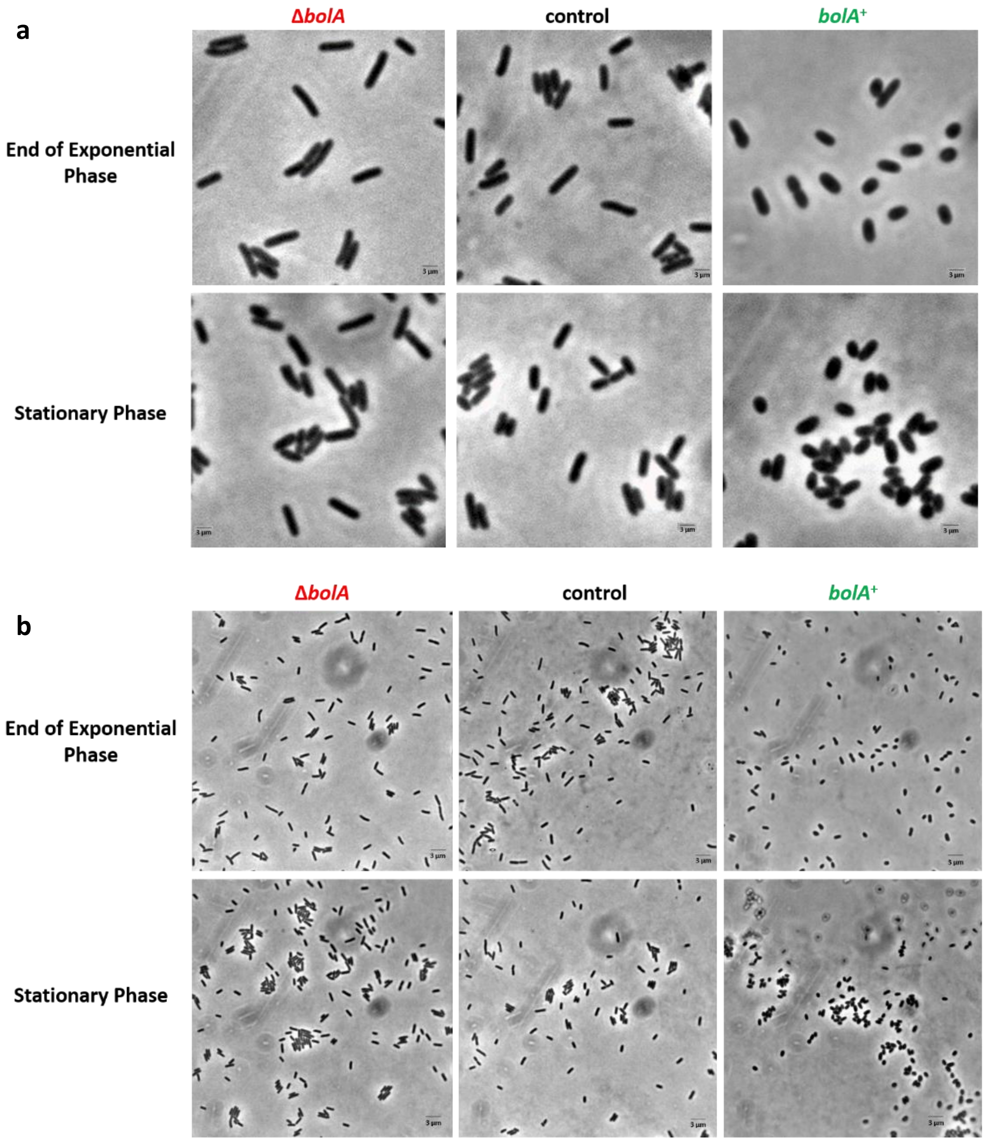

Fig. S2

The morphology of S. Typhimurium at the time points of cell collection. Bright-field microscopy images of S. Typhimurium SL1344 WT-Pwsk29 (control), Δ*bolA* and *bolA*$^+$ strains acquired at the end of exponential (5 hours) and stationary phases (8 hours) in LPM medium. **a)** Zoomed in sections of the original microscopy images show, as expected, that the strain *bolA*$^+$ has a more spherical morphology when compared to the strains control and Δ*bolA*. This observation confirms that BolA is being overexpressed at the time cells are collected for metabolomics analysis. No significant differences in morphology can be seen between the control and Δ*bolA* strains. **b)** Original microscopy images.

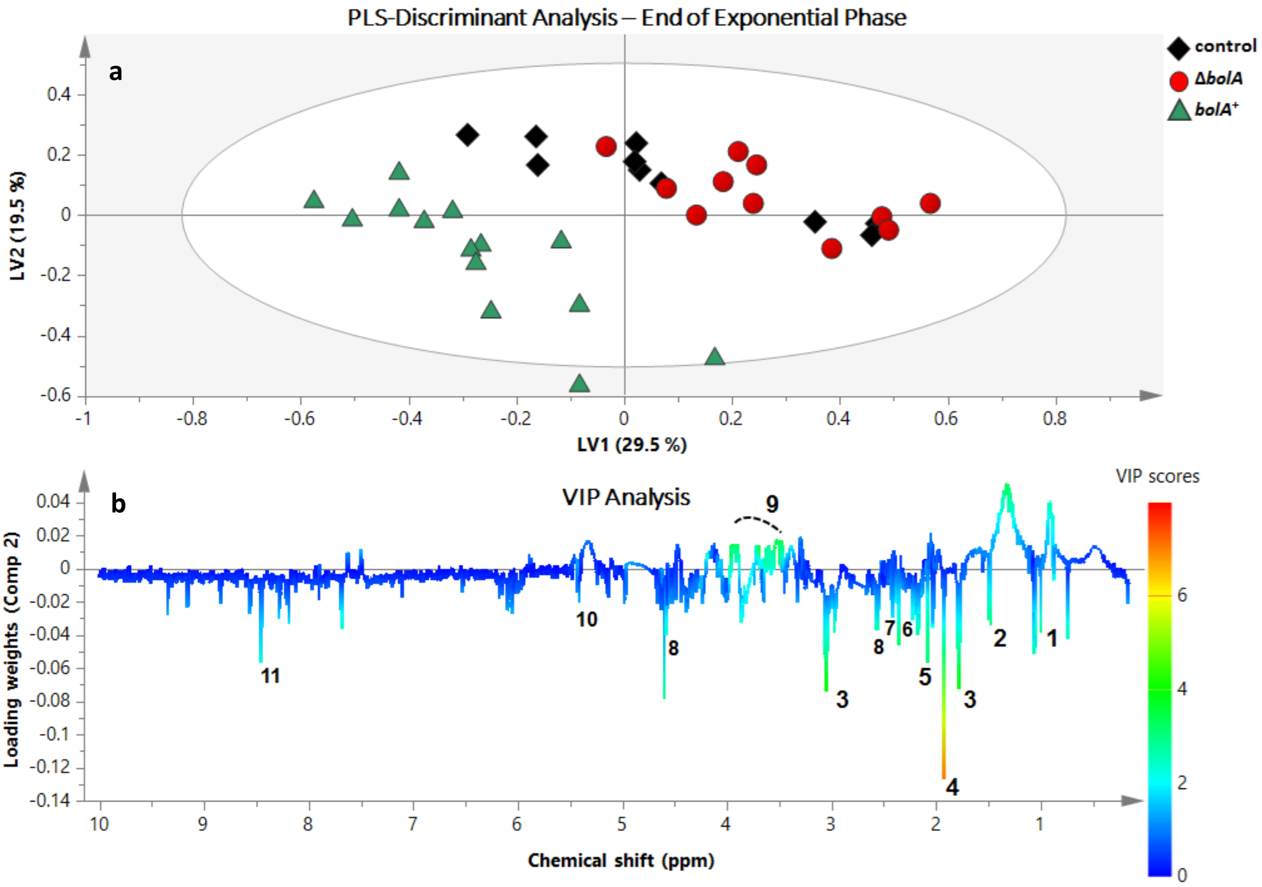

Fig. S3

PLS-DA model and VIP analysis of the control, Δ*bolA* and *bolA*$^+$ strains in the end of the exponential phase. **a)** Scores scatter plot of PLS-DA model of control (n=10), Δ*bolA* (n=11) and *bolA*$^+$ (n=14) samples in the end of exponential phase (R^2X=0.707, R^2Y=0.832, Q^2=0.599). **b)** VIP analysis of loading weights derived from component 1 of the PLS-DA model reveals the metabolites that contribute the most to the discrimination observed in the scores scatter plot. Legend: 1 – valine; 2 – alanine; 3 – putrescine; 4 – acetate; 5 – glycoprotein N-acetyl groups; 6 – unknown compound (2.2 ppm); 7 – succinate; 8 – glutathione; 9 – unknown(s); 10 – glycogen; 11 – formate.

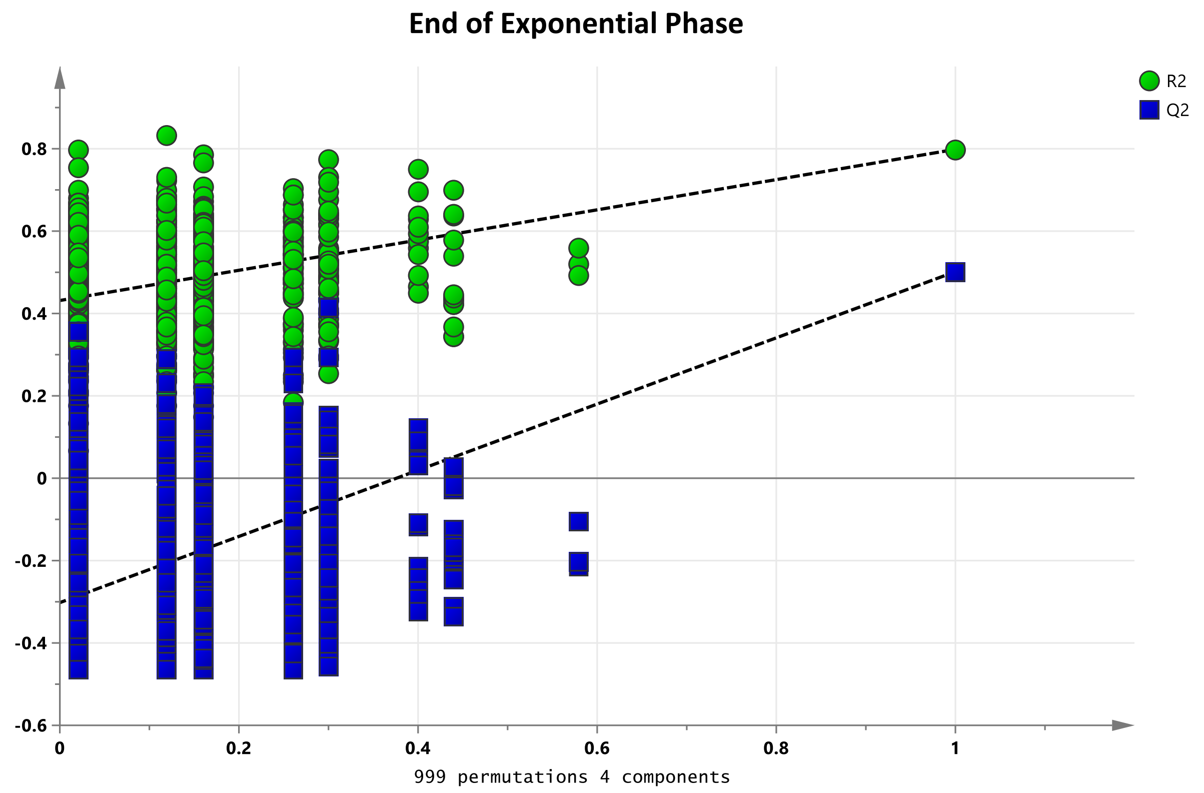

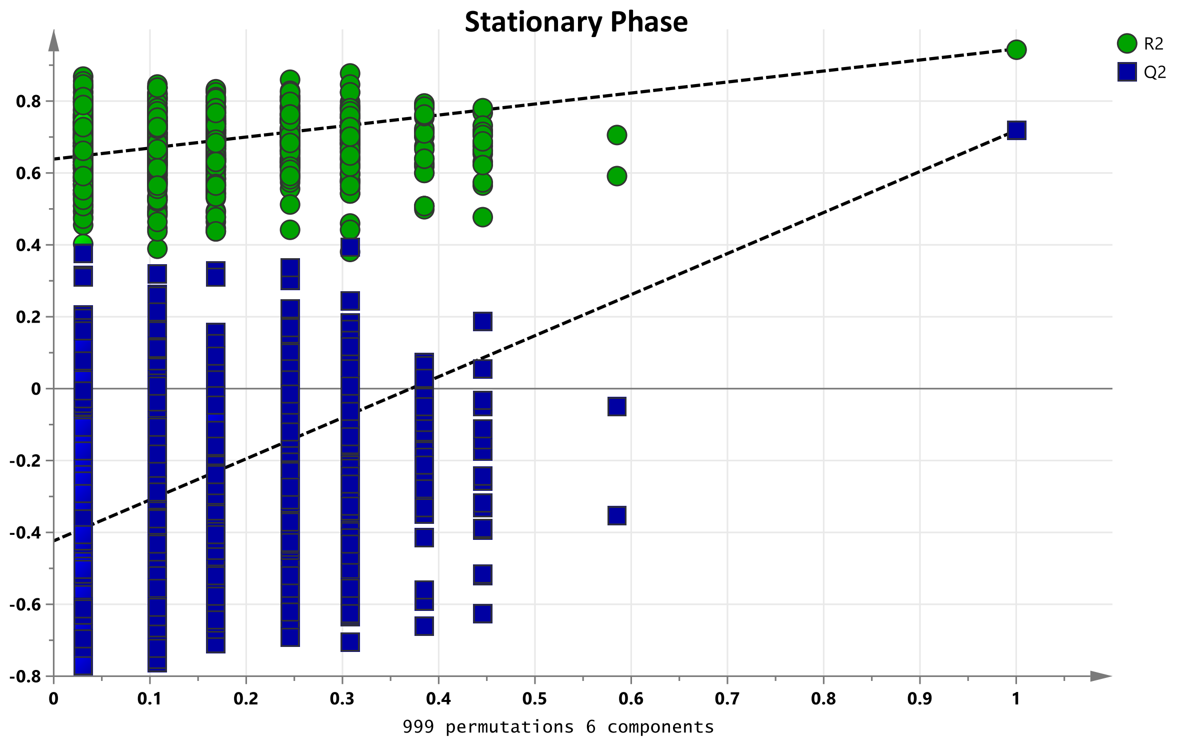

Fig. S4

Validation of the PLS-DA models. Permutation analyses (999 rounds) were performed for the PLS-DA models of the EE and ST growth phases. **a)** Permutation analysis of the EE phase resulted in models with Q^2 values inferior to those of the original model ($Q^2 = 0.599$). **b)** Permutation analysis of the ST phase resulted in model with Q^2 values inferior to those of the original model (Q^2=0.758).

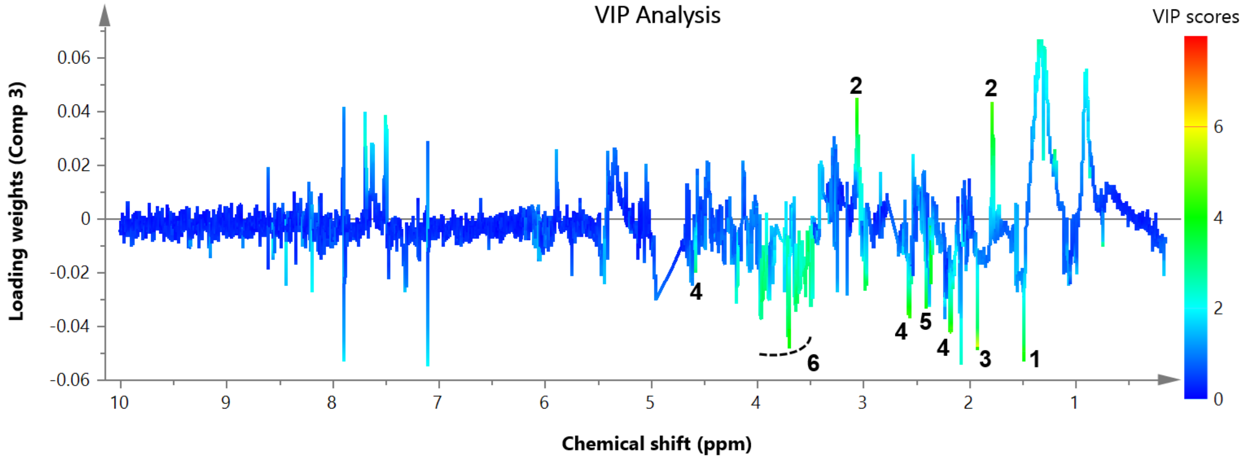

Fig. S5
VIP analysis of loading weights derived from component 3 of the PLS-DA model (stationary phase) reveals the metabolites that contribute the most to the discrimination between the control and Δ*bolA* strains, observed in the scores scatter plot. Legend: 1 – alanine; 2 – putrescine; 3 – acetate; 4 – glutathione; 5 – succinate; 6 – unknown(s)

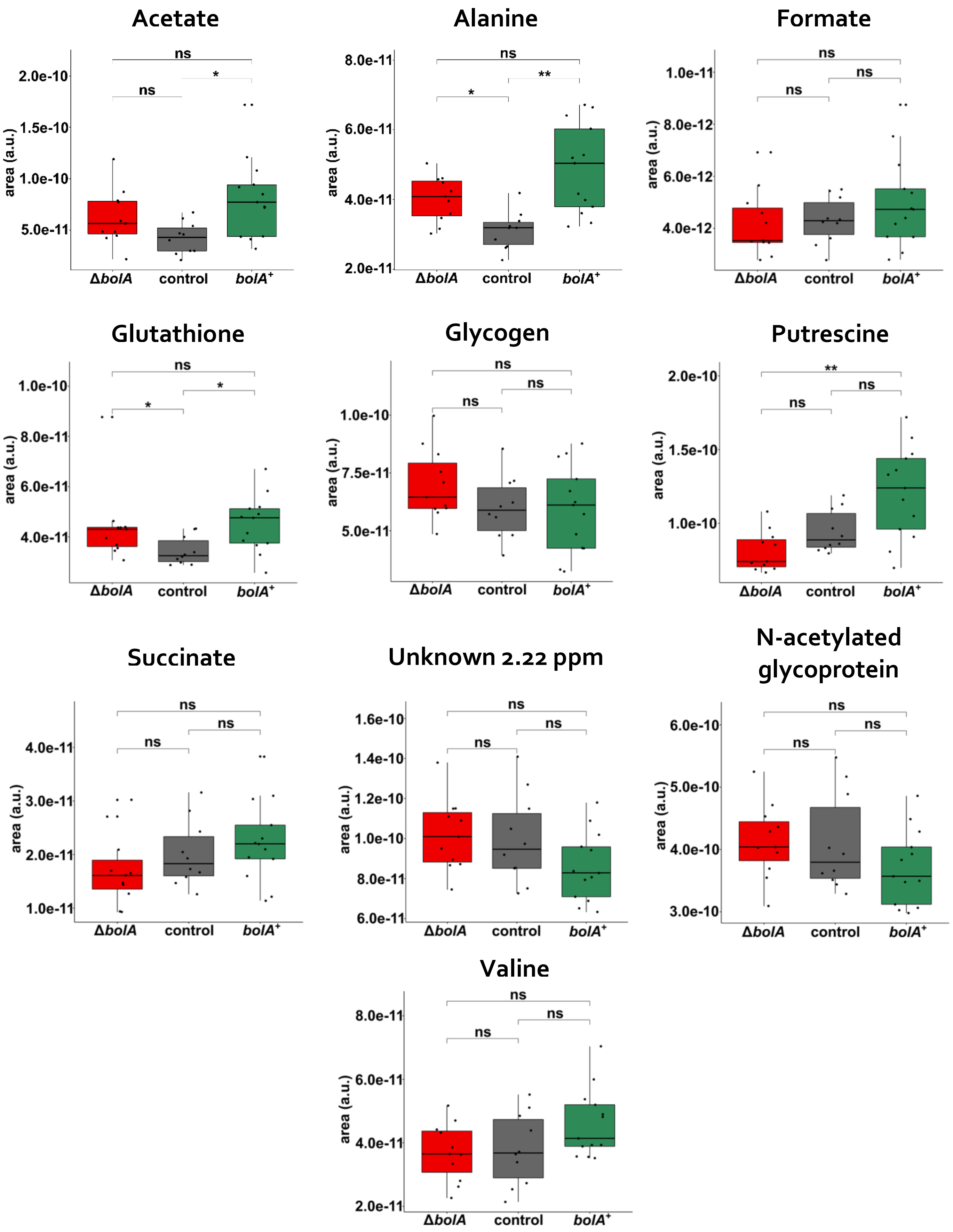

Fig. S6

Univariate analysis of the metabolites found to be altered in the EE growth phase. Variation of the metabolites found in the VIP analysis between the control, Δ*bolA* and *bolA*$^{+}$ strains. p > 0.05 (ns); p <= 0.05 (*); p <= 0.01 (**).
